# The gliadin peptide 31-43 exacerbates kainate neurotoxicity in epilepsy models

**DOI:** 10.1038/s41598-017-14845-4

**Published:** 2017-11-09

**Authors:** Elisabetta Gerace, Francesco Resta, Elisa Landucci, Daniela Renzi, Alessio Masi, Domenico E. Pellegrini-Giampietro, Antonio Calabrò, Guido Mannaioni

**Affiliations:** 1 Department of Health Sciences, Section of Clinical Pharmacology and Oncology, Viale G. Pieraccini 6, 50139 Florence, Italy; 2 Department of Neuroscience, Psychology, Drug Research and Child Health (NeuroFarBa), Viale G. Pieraccini 6, 50139 Florence, Italy; 3 Department of Experimental and Clinical Biomedical Sciences, University of Florence, Viale Morgagni 50, 50141 Florence, Italy

## Abstract

Many neurological disorders of gluten-related diseases (GRD), not directly referable to the gastrointestinal tract, have been reported in association with celiac disease (CD), including ataxia, neuropathy and epilepsy. In particular, people with epilepsy diagnosed with CD seems to be characterized by intractable seizure. In these patients, gluten restriction diet has resulted in a reduction of both seizure frequency and antiepileptic medication. Many hypotheses have been suggested, however, molecular mechanisms that associates GRD and epileptogenesis are yet unknown. In this study, we examined the effects of the toxic gliadin peptide 31-43 in *in vivo* and *in vitro* models of kainate-induced-epilepsy. We observed that p31-43 exacerbates kainate neurotoxicity in epilepsy models, through the involvement of the enzymatic activity of transglutaminases. Moreover, electrophysiological recordings in CA3 pyramidal neurons of organotypic hippocampal slices show that p31-43 increases the inward current induced by kainate, the average sEPSC amplitude and the total number of evoked action potentials when applicated alone, thus suggesting that p31-43 is able to influence CA3-CA1 neurotransmission and can potentiate postsynaptic kainate receptors. Our results suggest a possible mechanism underlying the relationship between GRD and epilepsy through a potentiation of kainate-induced neurotoxicity and links the toxic effects of gluten to epilepsy.

## Introduction

The symptoms of Celiac Disease (CD) or gluten-related diseases (GRD) are mostly characterized by digestive symptoms, which are often the first perceived signs of gluten intolerance. However, converging evidences suggest that the gluten-mediated immune response is frequently associated with neurological and psychiatric manifestations, including ataxia, neuropathy and epilepsy^1^. In particular, people with epilepsy seem to be diagnosed with CD far more often than the general population. Data suggest that up to 22% of patients with CD develop neurologic or psychiatric dysfunction^2^, and as many as 57% of people with neurological dysfunction of unknown origin test positive for anti-gliadin antibodies^3^. It is interesting to notice that in “intractable epilepsy”, gluten restriction diet has resulted in a reduction of seizure frequency with a decrease in antiepileptic medication needed to control intractable seizure in humans^4^. Unfortunately, there is no evidence on the casual relation between epilepsy and GRD and it remains unclear whether gluten contributes to the pathogenesis of these disorders or whether it represents an epiphenomenon. There are many hypotheses to explain this correlation and possible mechanisms have been suggested, such as: autoimmune mechanisms^5^, malabsorption^6^ and gluten toxicity^7^.

Transglutaminases (TGs) are a calcium-dependent enzyme of the protein-glutamine γ-glutamyltransferases family which catalyze the cross linking of a glutaminyl residue to a lysyl residue. To date, at least eight different human TGs have been identified and recently their roles in several diseases have been investigated^8^. TG2, particularly notable for being the autoantigen in CD, is the most widely expressed and abundant member of the transglutaminase family^9^. The anti-TG2 antibodies are used as serological tests for identifying CD patients, but have been recently implicated in the pathogenesis of several neurodegenerative diseases^10^. Another enzyme of the transglutaminase family primarily expressed in the central nervous system is transglutaminase 6 (TG6)^11^. Specifically, it was reported that anti-TG6 antibodies are gluten-dependent and appear to be a specific marker for neurologic manifestations. Indeed, TG6 antibodies were identified in immune-mediated ataxia in patients with gluten sensitivity thus suggesting a critical role for TG6 in cortical and cerebellar neurons^12^.

The gliadin peptide 31-43 (p31–43), is one of the main gliadin peptides that remain undigested by the intestine^13^ and has been shown to be toxic both in *in vitro* and *in vivo* tissues obtained from patients with CD^14,15^. Moreover, the p31–43 is able to initiate both a stress and an innate immune response with interleukin-15 (IL-15) as a major mediator in celiac intestine^16^. However, the effect and the molecular mechanisms of toxicity induced by p31-43 in the brain are poorly understood.

In this research, we studied the effects of gliadin peptide 31-43 in *in vivo* and *in vitro* models of kainate-induced-epilepsy and we observed that p31-43 exacerbates kainate neurotoxicity in epilepsy models, thus suggesting an increased excitotoxic synaptic activity of CA3-CA1 neurotransmission. Furthermore, we evaluated the involvement of the enzymatic activity of transglutaminases.

## Results

### The gliadin peptide p31-43 aggravates the seizure behavior in an *in vivo* model of temporal epilepsy

**Figure 1:**
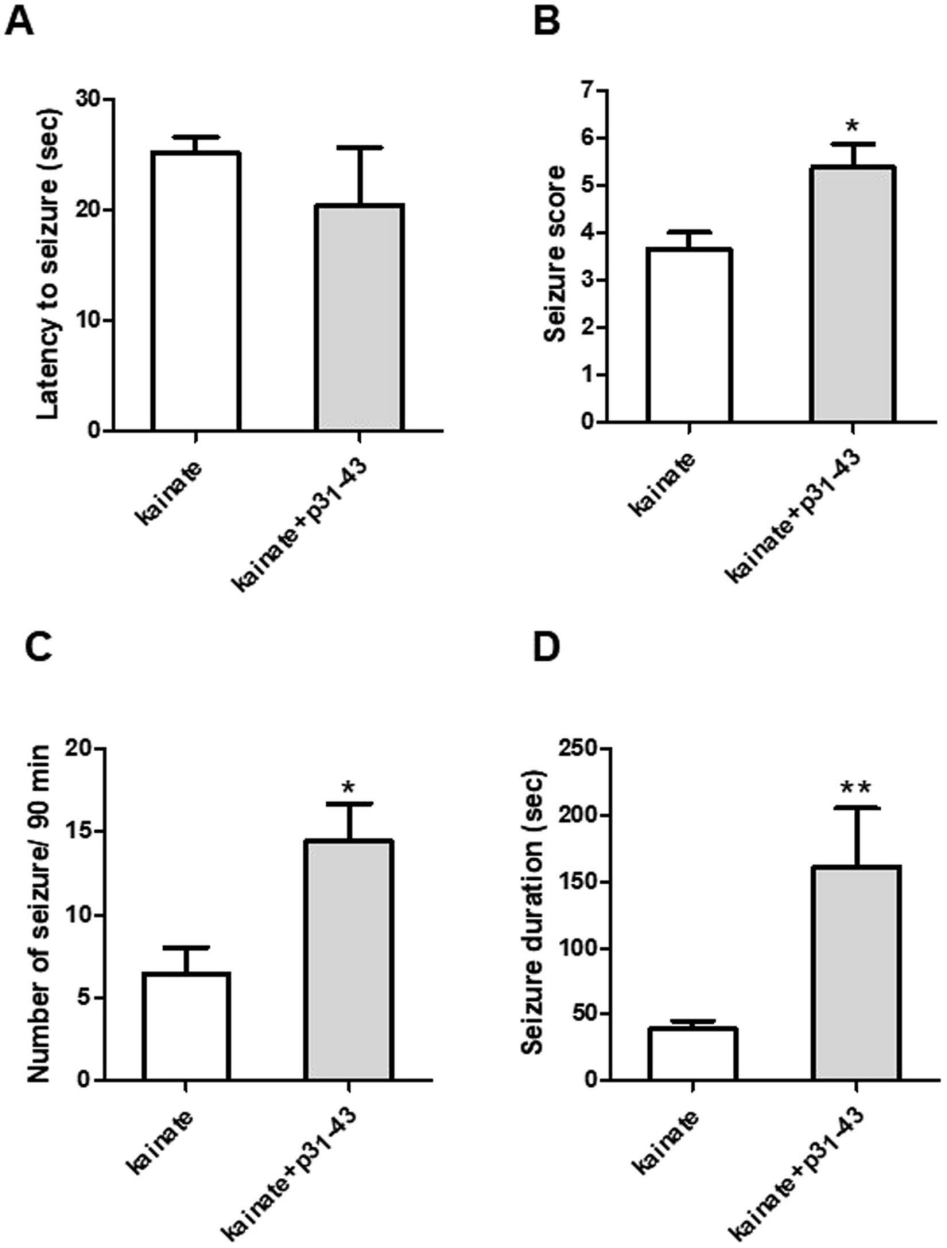
Exacerbation of kainate-induced seizure behavior induced by the gliadin peptide p31-43 in an *in vivo* model of temporal epilepsy. C57/B mice (9 per group) were injected i.p. with kainate (30 mg/kg, i.p.) or with kainate + p31-43 (30 mg/Kg). The seizure behavior test, including latency (**A**), score (**B**), number (**C**) or duration (**D**) of seizures, was performed for 90 min after kainate injection. The administration of p31-43 enhanced the seizure score, the number and duration of seizures induced by kainate. The bars represent the mean ± S.E.M. of three independent experiments. *p < 0.05 and **p < 0.01 vs. kainate (Student’s paired *t* test).

C57/B mice were injected (i.p.) with kainate (30 mg/k), p31-43 (30 mg/kg) alone or with the combination of them and the animals were observed for 90 min in order to assess latency, type and duration of epileptic seizures. p31-43 is not convulsive *per se* (data not shown), in contrast, the administration of p31-43 exacerbates the number and the duration of seizures induced by kainate (Fig. 1B–D) without changing the latency (Fig. 1A). Moreover, we observed that animals treated with p31-43 and kainate combination presented a higher mortality rate at the end of the experiments (44%) compared to animals treated with kainate alone (22%), thus demonstrating the noxious effects of p31-43 on kainate-induced seizure.

### The gliadin peptide p31-43 increases kainate-induced inward current in CA3 pyramidal cells of organotypic hippocampal slices

**Figure 2:**
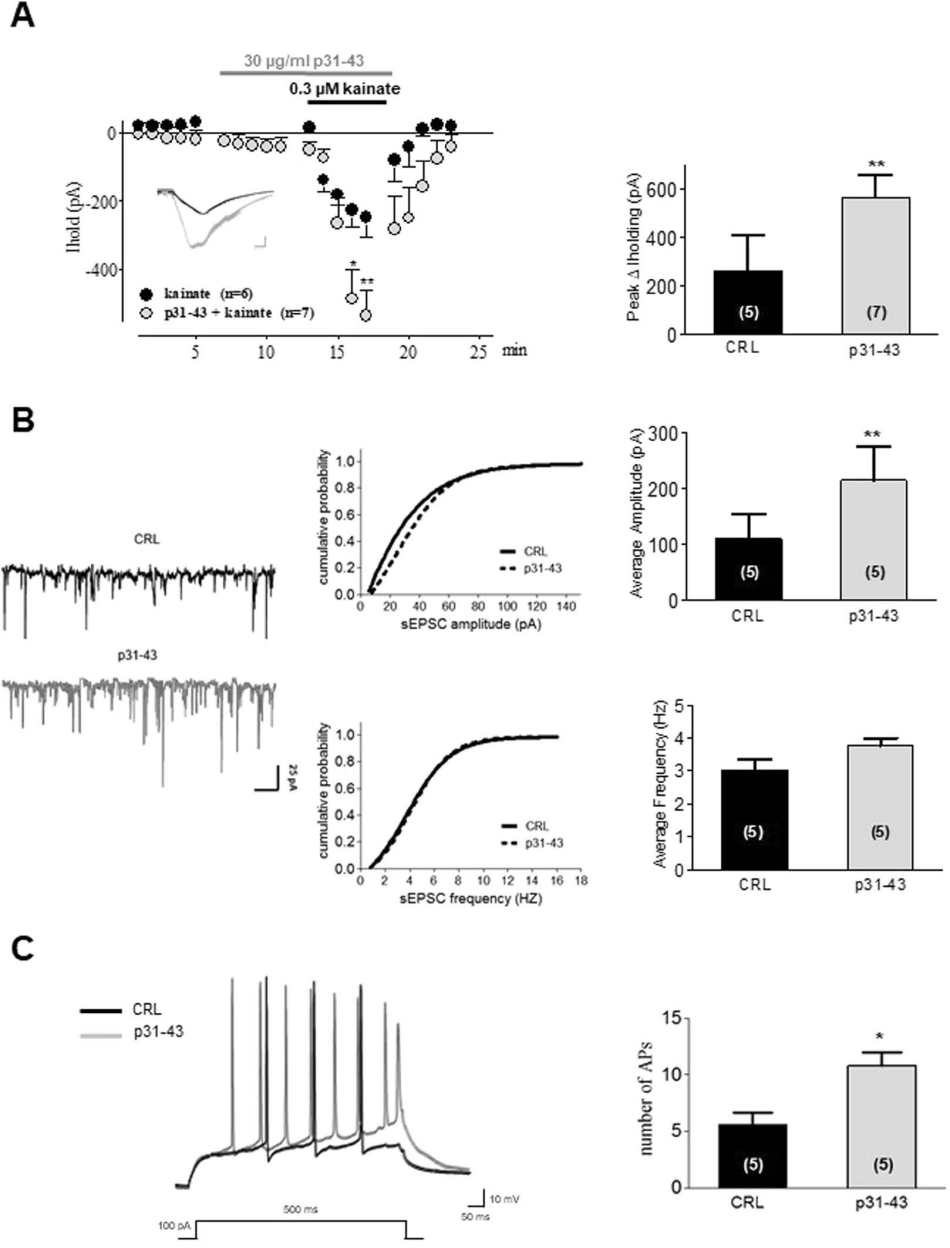
Effects of p31-43 on kainate-induced inward current in CA3 pyramidal cells of organotypic hippocampal slices. (**A**) *Left panel*: time course of kainate-stimulated inward current in CA3 pyramidal cells. Kainate (black circles) 0.3 µM induced an inward current that was enhanced by the pre-incubation with p31-43 (30 µg/ml for 5 min, grey circles). Example of a filtered typical trace showing a kainate-induced inward current and its increase induced by co-application of p31-43 is reported in the inset (scale bar: 100 pA/1 min). *Right panel*: Bar graph showing the mean current peak values in kainate and kainate + p31-43 treated slices. Data are the mean ± SEM, *p < 0.05, **p < 0.01 vs. control (CRL) (1 way ANOVA + Tukey’s *w* test). (**B**) *Left panel*: spontaneous excitatory postsynaptic currents (sEPSCs) recorded in whole-cell voltage-clamp configuration from CA3 pyramidal cells in control and during application of p31-43. Traces are representative of results obtained in five independent neurons for each condition. *Middle panel*: the application of p31-43 caused a significant shift in the cumulative probability plots on sEPSC amplitude *(top)* but not on sEPSC inter-event interval (*bottom*) [Kolmogorov-Smirnov (K-S) statistics, p < 0.01 vs. CRL]. *Right panel:* bar chart of quantitative data expressed as percentage of control amplitude (pA) showing that p31-43 significantly increased the amplitude but not the frequency of sEPSCs. Bars represent the mean ± SEM, **p < 0.01 vs CRL (Student’s paired *t* test). (**C**) *Left panel*: example of whole-cell current-clamp action potentials (APs) recorded from CA3 pyramidal cells evoked by a depolarizing single step (100 pA), before and after application of p31-43. *Right panel*: bar chart showing the increase of the total AP number in p31-43 treated neurons. Bars represent the mean ± SEM, *p < 0.05 vs CRL (Student’s paired *t* test). Number of cells is in parenthesis.

Our *in vivo* results suggest that p31-43 could potentiate the kainate-induced neurotoxicity on CA3 hippocampal neurons. In order to demonstrate our hypothesis, we performed electrophysiological experiments by recording kainate-induced inward current in CA3 pyramidal cells of organotypic hippocampal slices. We observed that p31-43 (30 µg/ml, pre-incubated for 5 min) increases the inward current induced by kainate (0.3 µM) (Fig. 2A), indicating an enhanced excitability during kainate stimulation that could explain our *in vivo* results. We then analyzed the electrophysiological responses of p31-43 alone on spontaneous excitatory synaptic currents (sEPSCs). We exposed CA3 neurons to p31-43 (30 µg/ml) for 5 min and we measured the amplitude and the frequency of sEPSCs during its application. We observed that p31-43 caused a significant shift in the cumulative probability plots on sEPSC amplitude (Fig. 2B middle panel top) but not on sEPSC inter-event interval (Fig. 2B middle panel bottom). Moreover, p31-43 caused a significant increase of sEPSC average amplitude calculated for each cell (Fig. 2B, *top* middle panel). No effect was observed on the sEPSC frequency (Fig. 2B right panels). In addition, we measured number, latency, amplitude and threshold of evoked action potentials (APs) to study the effect of p31-43 on the overall intrinsic neuronal excitability. Figure 2C shows that the total number of evoked APs was significantly increased in neurons treated with p31-43. No effects of p31-43were observed on resting membrane potentials (RMP, firing activity) (data not shown). We evaluated the possibility that the increased neuronal excitability could be caused by an alteration of the passive membrane properties. To this aim, we measured the membrane resistance and the membrane capacitance before and after the application of p31-43. We observed that neither the membrane resistance (baseline: 121,8 ± 9,961 MΩ, n = 5; p31-43: 117,0 ± 12,74 MΩ, n = 5; p = 0.60) nor the membrane capacitance (baseline: 263,8 ± 11,77 MΩ, n = 5; p31-43: 266,5 ± 22,31 MΩ, n = 5; p = 0.18) were significantly changed after the application of p31-43, thus excluding an effect on passive cell membrane properties.

### The gliadin peptide p31-43 exacerbates kainate neurotoxicity in an *in vitro* model of epilepsy

**Figure 3:**
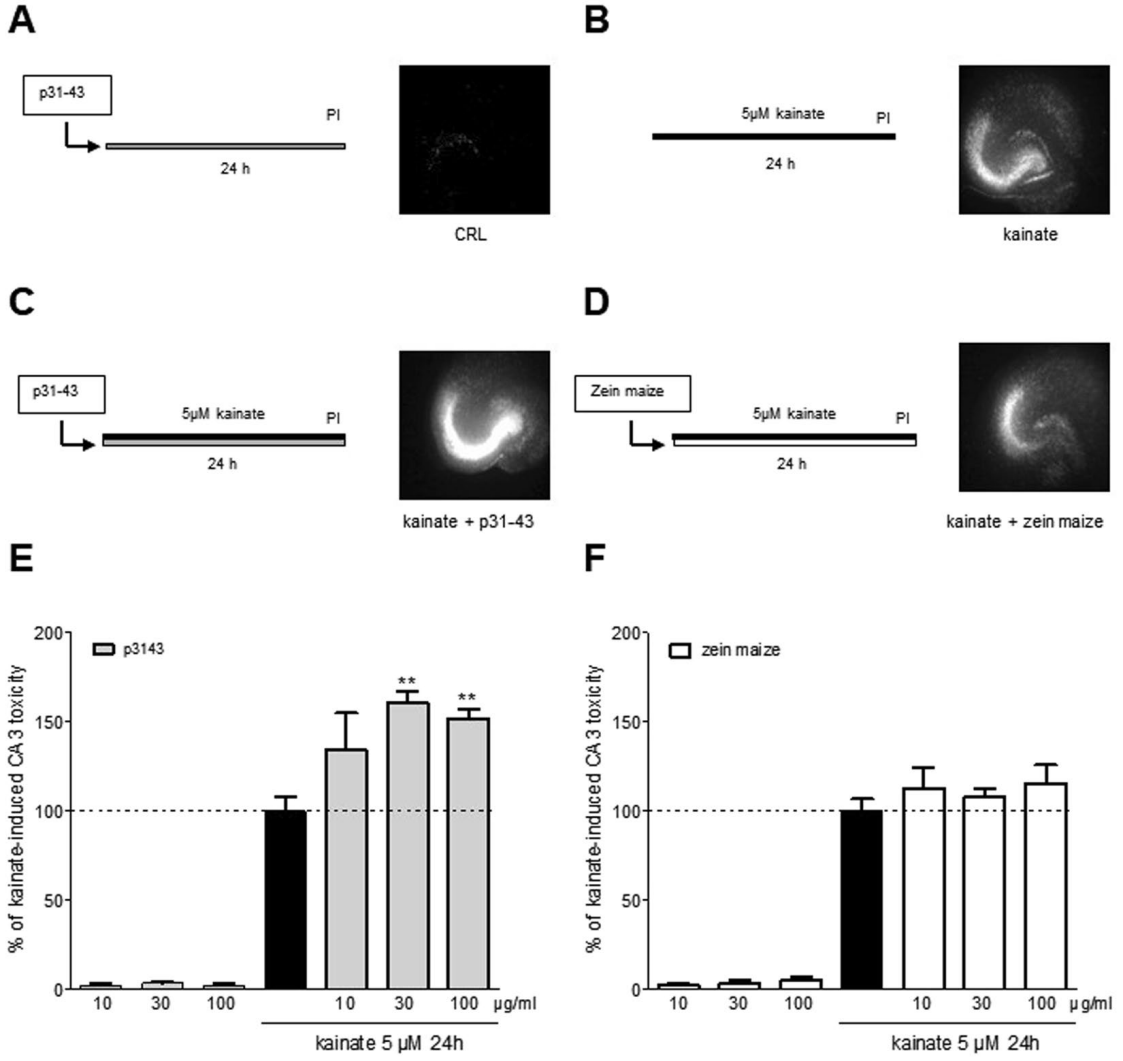
Exacerbation of kainate-induced neurotoxicity by the gliadin peptide p31-43 in an *in vitro* model of epilepsy. (**A–D**) Rat organotypic hippocampal slices were incubated for 24 h with p31-43 (30 µg/ml) (**A**), 5 µM kainate (**B**), kainate + 30 µg/ml p31-43 (**C**) or kainate + 30 µg/ml maize zein and then incubated with propidium iodide (PI) and observed under fluorescence optics to detect neuronal injury. Kainate toxicity in the CA3 region was exacerbated by p31-43 but not maize zein. (**E**) Quantitative analysis is expressed as percentage of damage produced by 5 µM kainate. Incubation with p31-43 or maize zein alone was not toxic; p31-43 (30-100 µg/ml) but not maize zein significantly enhanced kainate injury. Bars represent the mean ± SEM of al least five experiments. **p < 0.01 vs. kainate (ANOVA + Tukey’s *w* test).

It is still unclear whether a long exposure to p31-43 could be able to cause CA3 neurodegeneration. Therefore, we tested the chronic application of p31-43 in our *in vitro* model of epilepsy. To this aim, we exposed organotypic hippocampal slices to 5 µM kainate for 24 h. Under these conditions, the slices underwent selective injury of the CA3 pyramidal cells (Fig. 3B), which is considered a classical model of temporal epilepsy^17^. We added p31-43 (10-100 µg/ml) to the incubation medium alone (Fig. 3A) or during 24 h of kainate exposure (Fig. 3C). Accordingly with our previous results, p31-43 do not induce toxicity by itself, when incubated alone for 24 h (Fig. 3A), while it exacerbates CA3 injury induced by kainate in a dose-dependent manner (Fig. 3E, *left panel*). Conversely, the incubation with maize zein, a prolamin protein derived from corn used as control peptide, was not toxic by itself and did not alter kainate-induced CA3 toxicity (Fig. 3E, *right panel*), thus suggesting that specifically p31-43 was responsible for increased kainate induced-damage.

### The gliadin peptide p31-43 exacerbates kainate neurotoxicity through the involvement of transglutaminases in organotypic hippocampal slices

**Figure 4:**
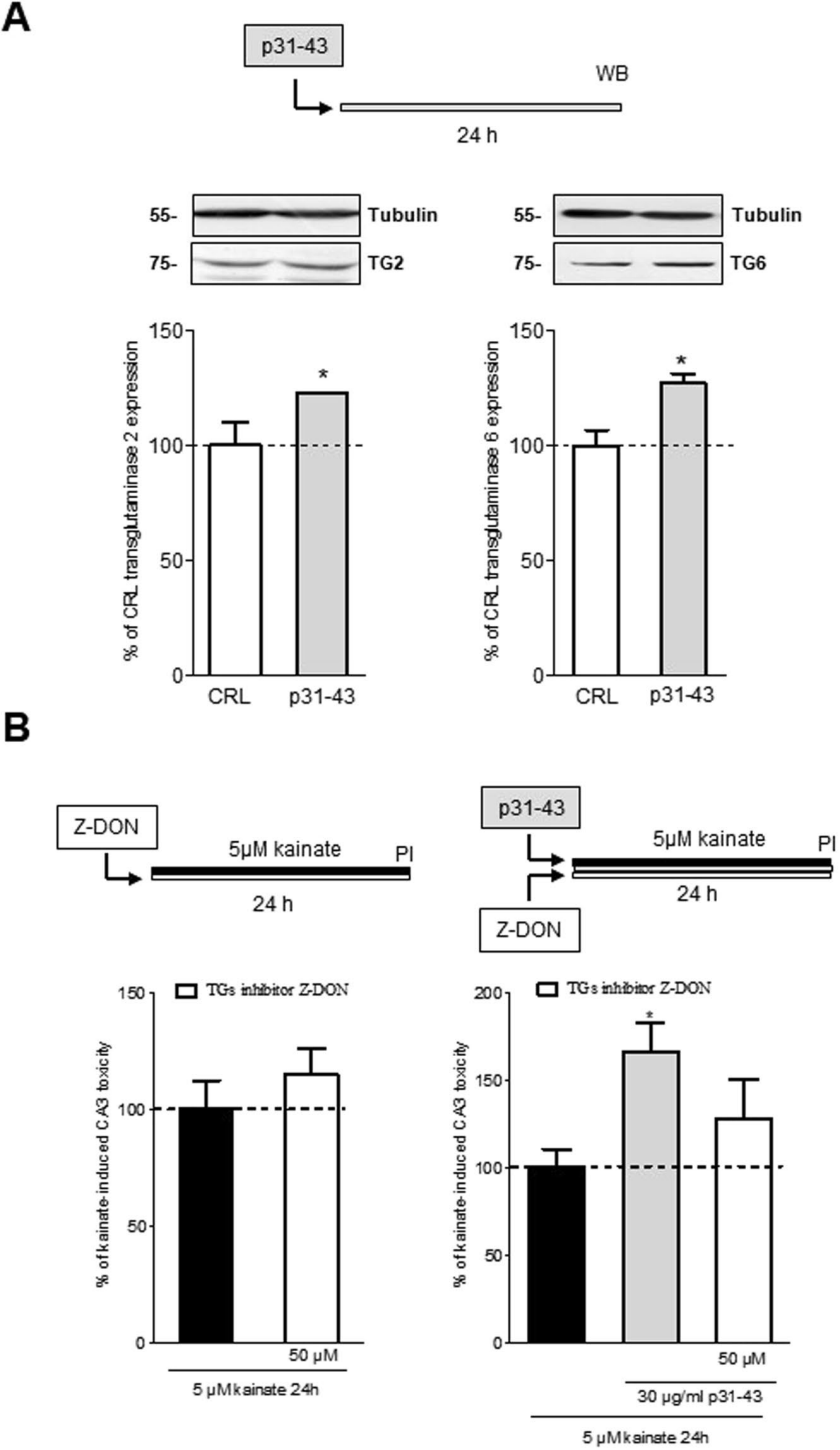
Involvement of transglutaminases in the neurotoxicity induced by the gliadin peptide p31-43 in an *in vitro* model of epilepsy. (**A**–**D**) Rat organotypic hippocampal slices were incubated for 24 h with p31-43 (30 µg/ml) (**A**) and analysed by western blotting. (**A**) Incubation with p31-43 for 24 h significantly increased the expression of transglutaminase (TG) 2 (100 ± 10 vs 122 ± 0.08) and TG6 (100 ± 6.7 vs 127 ± 4.23) in organotypic hippocampal slices, as detected by Western blot using polyclonal anti-TG2 or anti-TG6 antibodies. Data are expressed as percentage of control TG2 or TG6 levels. Bars represent the mean ± SEM of 3 experiments. *p < 0.05 vs. control TG2 or TG6 (ANOVA + Tukey’s *w* test). Qualitative images of blots is representative of densitometric analysis and show blots cropped from different parts of the same gel. The full-length gels and blots are included in Fig. 4a and Fig. 4b of the Supplementary Information. (**B**) The addition of the TG inhibitor Z-DON (50 µM) was not toxic by itsef (*left*) but attenuated kainate + p31-43 CA3 toxicity (*right*) in organotypic hippocampal slices. Data are expressed as percentage of kainate toxicity. Bars represent the mean ± SEM of 4 experiments. (ANOVA + Tukey’s *w* test).

We hypothesize that the toxic effects of p31-43 on kainate-induced neurotoxicity could be mediated by transglutaminases involvement. Therefore, we studied the expression level of TG2 and TG6 proteins after exposure to p31-43 by western blotting technique in homogenates of organotypic hippocampal slices. We observed that the incubation with p31-43 (30 µg/ml for 24 h) significantly increased the expression of TG2 and TG6 (Fig. 4A). Therefore, we tested the non-selective TGs inhibitor Z-DON-Val-Pro-Leu-OMe (Z-DON) as a possible neuroprotective drug in our system. We exposed hippocampal slices for up to 24 h to Z-DON (50 µM) plus kainate (5 µM) alone or in combination with p31-43 (30 µg/ml) and then we evaluated CA3 pyramidal cell injury with PI fluorescence. The addition of Z-DON was not neuroprotective on kainate alone (Fig. 4B, *left panel*) but attenuated kainate plus p31-43 CA3 induced-toxicity (66 ± 16% and 28 ± 22%, respectively) (Fig. 4B, *right panel*).

## Discussion

The present study reports, for the first time, the toxic effects of the gliadin peptide 31-43 on kainate-induced seizures in an *in vivo* model of epilepsy. Our results show that the gliadin peptide p31-43 does not provoke convulsions per se, while the administration of p31-43 in C57/B mice exacerbates the number and the duration of seizures induced by kainate. In addition, the animals treated with the combination of p31-43 and kainate presented a higher mortality at the end of the experiments compared to animals treated with kainate alone, thus demonstrating the noxious effects of p31-43 on kainate-induced seizure. Our data reproduce what has been widely observed in many clinical case reports that describe a link between epilepsy and CD^18^. In particular, a specific type of focal epilepsy associated with occipital calcifications was described by Gobbi and co-workers^19^. This form is characterized by antiepileptic drug-resistant seizures and has been shown to benefit from gluten restriction diet^4^. Since no animal model mimicking all the key factors predisposing to CD (i.e. intestinal epithelial barrier dysfunction, sensitization of CD4+ T cells, chronic exposure to dietary gluten etc.) is currently available, in our *in vivo* experiments we injected p31-43 i.p. in order to cross the intestinal barrier thus mimicking the altered intestinal permeability of CD.

To explain the results obtained in our experiments *in vivo*, we hypothesized that p31-43 could potentiate the kainate-induced neurotoxicity on CA3 hippocampal neurons, which are the main targets of kainate in the hippocampal region (because of the high density of KA1 receptors in the CA3 that could explain the selective vulnerability of these neurons to kainate^20^. For this reason, we recorded kainate-induced inward currents in CA3 pyramidal cells of organotypic hippocampal slices during the application of p31-43 alone or incubated with kainate. Our data show that p31-43 increases the inward current induced by kainate, indicating an enhanced excitability during kainate stimulation and supporting our *in vivo* results. Furthermore, the average sEPSC amplitude during the 5-min p31-43 application was significantly increased (195 ± 27% of the control (n = 7, *p* < 0.001)) as well as the shift in the cumulative probability plots on sEPSC amplitude. These findings indicate that the gliadin peptide induces postsynaptic modifications and suggest that p31-43 can potentiate glutamatergic synaptic transmission influencing the sensitivity of postsynaptic kainate receptors. Additionally, p31-43 provokes also a significant increase in the total number of evoked APs, suggesting that p31-43 is able to increase neuronal excitability that could result in enhanced firing probability following an excitatory stimulus, such as kainate-induced depolarization.

Considering that it is still unclear whether a long exposure to p31-43 could be able to cause CA3 neurodegeneration, we tested the chronic application of p31-43 in our *in vitro* model of kainate induced CA3 toxicity, which is considered a classical model of temporal epilepsy^17^. p31-43 do not induce toxicity by itself, when incubated alone for 24 h, while it exacerbates CA3 injury induced by kainate in a dose-dependent manner. Conversely, the incubation with maize zein, a prolamin protein derived from corn used as control peptide, was not toxic by itself and did not alter kainate-induced CA3 toxicity, thus suggesting that specifically p31-43 was responsible for increased kainate induced-damage.

We hypothesized that the toxic effects of p31-43 on kainate-induced neurotoxicity could be mediated by transglutaminases involvement. The role of TG2 in CD has now very well established exerting at least two crucial roles: as a deamidating enzyme of gliadin peptides, that can enhance the immunostimulatory effect of gluten and as a target autoantigen in the immune response^9^. Glutamine-rich gliadin peptides are excellent substrates for TG2. Indeed, up to 36% of the glutamine (Gln) residues in gliadin were accessible to modification by TG2. The physiological roles of TG2 are not fully characterized and many basic questions remain unresolved. Conversely, TG2 has been mostly linked to a number of diseases, including inflammatory bowel disease, cancer^21^, human immunodeficiency virus (HIV) infection^22^ and neurological disorders^10^. The TG6 isoform appears to be a specific marker for neurologic manifestations and recently it has been shown that neurodegenerative diseases, such as Alzheimer’s disease, Parkinson’s disease, Huntington’s disease and other polyglutamine diseases are characterized in part by aberrant cerebral transglutaminase activity and by increased cross-linked proteins in affected brains^23^. To explain our results, we theorized that that two different mechanisms in different time points might exist. Indeed, the early responses are probably due to post-synaptic kainate receptors potentiation induced by p31-43 leading to the observed electrophysiological effects (Fig. 2B). These effects are very fast and probably do not involve TGs modification. On the other hand, kainic acid application induces a neuronal damage and a possible lower of pH. In these conditions, TGs enzymes induce an increase in glutamate residues by glutamine deamidation of p31-43 (Di Sabatino *et al*.^9^) that may contribute to the potentiation of kainate toxicity. Therefore, we studied the expression level of TG2 and TG6 proteins after exposure to p31-43 by western blotting technique in homogenates of organotypic hippocampal slices. We observed that the incubation with p31-43 significantly increased the expression of TG2 and TG6, supporting our hypothesis according to which p31-43 may determine toxicity by increase glutamate residues by Gln deamination due to the activation or overexpression of TGs. Our theory is also reinforced by the results obtained with maize zein that is a prolamin protein that does not contain glutamine residues and does not provoke toxicity by itself nor alters kainate-induced CA3 toxicity. Our results are supported by Caputo and coworkers which observed that prolonged treatment of human intestinal Caco-2 cell line (extensively used as a model of the intestinal barrier) with p31-43 increased TG2 protein expression, indicating a non-tissue-specific post-translational mechanism of the gliadin peptide 31-43^24^. Furthermore, it has been shown that TG6 immunoreactivity is present in the CA3 region, but not in CA1/CA2 or in the dentate gyrus (DG)^25^ thus supporting our *in vitro* results in which we observed that p31-43 exacerbates kainate neurotoxicity selectively in the CA3 region of hippocampal slices. Hence, we tested the non-selective TGs inhibitor Z-DON-Val-Pro-Leu-OMe (Z-DON)^26^ as a possible neuroprotective drug in our system. The addition of Z-DON was not neuroprotective on kainate alone but attenuated kainate plus p31-43 CA3 induced-toxicity. Interestingly, TGs inhibitors were recently proposed for maintaining the integrity of intestinal tight junctions as a way of preventing downstream inflammatory cascades and are also effective in reducing the production of anti-TG2 antibodies when studied in duodenal biopsy cultures from untreated (gluten-containing diet) celiac patients^27^. Furthermore, a new possible role of TGs inhibitors in the therapy for Huntington’s disease^28^, Alzheimer’s disease^29^, Parkinson’s disease^30^, excitotoxicity and stroke^31,32^ has recently emerged.

In conclusion, our study associates the toxic effects of gluten to epilepsy. In particular, our findings suggest a possible mechanism underlying the relationship between gluten and epilepsy through a potentiation of neurotoxicity induced by kainate on CA3 hippocampal neurons by p31-43 and the overexpression and activation of neuronal TGs.

## Materials and Methods

Experiments and animal use procedures were in accordance with the National Institutes of Health Guide for the Care and Use of Laboratory Animals (NIH Publications No. 80-23, revised 1996). The experimental protocols were approved by the Animal Care Committee of the Department of Health Science, section of Pharmacology, University of Florence, in compliance with the European Convention for the Protection of Vertebrate Animals used for Experimental and Other Scientific Purposes (ETS no. 123) and the European Communities Council Directive of 24 November 1986 (86/609/EEC). The authors further attest that all efforts were made to minimize the number of animals used and their suffering.

## Materials

Kainic acid was purchased from Sigma (St Louis, MO, USA), synthetic peptide p31–43 (LGQQQPFPPQQPY) was purchased from Primm srl, (Milan, Italy), transglutaminases (TGs) inhibitor Z-DON (Z-DON-Val-Pro-Leu-OMe) was obtained from Zedira GmbH (Darmstadt, Germany). Propidium iodide (PI) was purchased from Sigma (St Louis, MO, USA). Tissue culture reagents were obtained from Gibco-BRL (San Giuliano Milanese, MI, Italy) and Sigma (St Louis, MO, USA).

## Methods

### ***In vivo*** model of epilepsy

We used the experimental protocol previously described by Muzzi *et al*.^32^. C57/Bl6 male mice (30 g) (Harlan Nossan, UK) were used for kainate-induced seizures. Seizure response was assessed by adopting the following scale previously described in Jiang *et al*., 2015: 0 = normal behavior, 1 = immobile and cleaved up posture, 2 = automatisms, 3 = partial body clonus and shivering, 4 = whole body clonus, rearing and falling, 5 = non intermittent seizure activity, 6 = wild running and tonic seizure, 7 = death. We measured the seizure score including latency to seizure, type and duration of seizures. Data are expressed as the mean ± SEM. All animal procedures were conducted according to the European Community Guidelines for Animal Care. C57/B mice (9 animals for each group) were randomly divided into 4 experimental groups in order to assess epileptic seizures induced by: *i)* kainate (single dose, 30 mg/kg, i.p.) (group 1), *ii)* p31-43 (single dose, 30 mg/kg, i.p.) (group 2), *iii)* kainate plus p31-43 (two single doses, 30 mg/kg, i.p.) (group 3) and *iv)* a comparable volume of 0.9% NaCl (group 4).

### Organotypic rat hippocampal slice model of epilepsy

Organotypic hippocampal slice cultures were prepared as previously reported^33,34^. Briefly, hippocampi were removed from the brains of 7- to 9-day old Wistar rat pups (Harlan, MI, Italy), transverse slices (420 µm) were prepared using a McIlwain tissue chopper and then transferred onto 30 mm diameter semiporous membranes inserts (Millicell-CM PICM03050; Millipore, Italy), which were placed in six well tissue culture plates containing 1.2 ml medium per well. The culture medium consisted of 50% Eagle’s minimal essential medium, 25% heat-inactivated horse serum, 25% Hanks’ balanced salt solution, 5 mg/ml glucose, 2 mM L-glutamine, and 3.75 mg/ml amphotericin B. Slices were maintained at 37 °C in an incubator in atmosphere of humidified air and 5% CO_2_ for two weeks. Before experiments all slices were screened for viability by incubating them for 30 min with PI (5 μg/ml); slices displaying signs of neurodegeneration were discarded from the study. After 2 weeks in culture, the slices were exposed to 5 µM kainate for 24 h. Under these conditions, the slices undergo selective injury of the CA3 pyramidal cells, which is considered a classical model of temporal epilepsy^17^. We have tested the effects of the gliadin peptide 31-43 (10-100 µg/ml) by adding the peptide to the incubation medium during the 24 h exposure to kainate.

### Assessment of CA1 pyramidal cell injury

PI (5 μg/ml) was added to the medium either at the end of the kainate incubation period. Thirty minutes later, fluorescence was viewed using an inverted fluorescence microscope (Olympus IX-50; Solent Scientific, Segensworth, UK) equipped with a xenon-arc lamp, a low-power objective (4X) and a rhodamine filter. Images were digitized using a video image obtained by a CCD camera (Diagnostic Instruments Inc., Sterling Heights, MI, USA) controlled by software (InCyt Im1^TM^; Intracellular Imaging Inc., Cincinnati, OH, USA) and subsequently analyzed using the Image-Pro Plus morphometric analysis software (Media Cybernetics, Silver Spring, MD, USA). In order to quantify cell death, the CA3 hippocampal subfield was identified and encompassed in a frame using the drawing function in the image software (ImageJ; NIH, Bethesda, USA) and the optical density of PI fluorescence was detected. There was a linear correlation between CA3 PI fluorescence and the number of injured CA3 pyramidal cells as detected by morphological criteria^35,36^.

### Electrophysiological recordings in organotypic hippocampal slices

The experiments were conducted as previously described in Gerace *et al*.^36^. Briefly, the organotypic hippocampal slices slices were placed into a recording bath submerged with ice-cold artificial cerebrospinal fluid (ACSF) containing (in mM): NaCl 130, KCl 3.5, Na_2_H_2_PO_4_ 3, NaHCO_3_, glucose, MgCl_2_ 1.5 and CaCl_2_ 1.5 at pH 7.4 and oxygenated with 95%O_2_/5%CO_2_. Recording microelectrodes were prepared from borosilicate glass (WPI Inc; Sarasota, FL) by a Narishige Instruments micropipette puller (Tujunga, CA) (resistance ranging from 3 to 5 MΩ) and filled with internal solution of the following composition (in mM concentrations): K-gluconate 142.5, potassium methylsulfate 20, NaCl 8, Hepes 10, EGTA 0.1, MgATP 2, and GTP 0.2. The pH of the internal solution was adjusted to 7.2 with KOH and the osmolarity was adjusted to 300 mOsm with H_2_O and sucrose. Recordings were done using a Multiclamp preamplifier (Axon Instruments; Foster City, CA) and filtered at 5 kHz. All the data were acquired, stored and analyzed on a PC using the pCLAMP (Axon Instruments, Foster City, CA, USA) and GraphPad softwares. Traces were filtered by a digital Gaussian filter (Clampfit facility, low pass, 200 Hz). The frequency and peak amplitude of detected events were analyzed using Mini Analysis Program (Synaptosoft Inc., www.synaptosoft.com, NJ). To evaluate the total synaptic input onto CA3 region, whole-cell voltage-clamp recordings were performed and spontaneous excitatory post-synaptic currents (sEPSCs) were recorded in CA3 region. Moreover, to study the effect of p31-43 on the whole intrinsic excitability, increasing steps of depolarizing current were imposed in current-clamp configuration to CA3 neurons to measure: number, latency, amplitude and threshold of evoked action potentials (APs).

### Western blot analysis

Cultured slices were washed with cold 0.01 M phosphate-buffered saline, pH 7.4 and 8 slices/sample were gently transferred and dissolved in a tube containing 1% SDS as previously reported^37^. Total protein levels were quantified using the Pierce (Rockford, IL, USA) BCA (bicinchoninic acid) Protein Assay. 40 μg of proteins were resolved by electrophoresis on a 8% SDS-polyacrylamide gel and transferred onto nitrocellulose membranes using the transblot TURBO (Bio-Rad, Hercules, CA, USA). Blots were probed overnight at 4 °C with the polyclonal rabbit transglutaminase-2 (TG2) (Zedira GmbH, Darmstadt, Germany) and transglutaminase-6 (TG6) (Abcam, Cambridge, UK), antibodies all diluted 1:1000. Immunodetection was performed with secondary antibodies (1:2000 anti-rabbit IgG from donkey (Amersham Biosciences, UK)) conjugated to horseradish peroxidase. The reactive bands were detected using chemiluminescence (ECLplus; Euroclone, Padova, Italy). Quantitative analysis was performed using the QuantityOne analysis software (Bio-Rad, Hercules, CA, USA).

### Statistics

Data are presented as means ± SEM of n independent experiments. Statistical significance was evaluated by unpaired Student’s t test, except for Kolmogorov-Smirnov (KS) statistics and PI fluorescence intensities that were evaluated by performing one-way ANOVA followed by Tukey’s w test for multiple comparisons. All statistical analysis were performed using Graph-Pad Prism v. 5 for Windows (GraphPad Software, San Diego, CA, USA). A probability value (P) of <0.05 was considered significant.

#### Study approval

All procedures were approved by the Animal Care Committee of the Department of Health Science, section of Pharmacology, University of Florence.

## Electronic supplementary material

Supplementary Dataset 1
