# Supplementary material for: The gliadin peptide 31-43 exacerbates kainate neurotoxicity in epilepsy models
Source: Sci Rep. 2017 Nov 9;7:15146. doi: 10.1038/s41598-017-14845-4 (PMC5680182; doi:10.1038/s41598-017-14845-4)
Supplement: Supplementary file 1 — Supplementary Dataset 1 [file 41598_2017_14845_MOESM1_ESM.pdf]

The gliadin peptide 31-43 exacerbates kainate neurotoxicity in epilepsy models.

[1,2]Elisabetta Gerace, [2]Francesco Resta, [1]Elisa Landucci, [3]Daniela Renzi, [2]Alessio Masi, [1]Domenico E. Pellegrini-Giampietro, [3]Antonio Calabrò & [2]Guido Mannaioni

[1]*Department of Health Sciences, Section of Clinical Pharmacology and Oncology, and* [2]*Department of Neuroscience, Psychology, Drug Research and Child Health (NeuroFarBa), Viale G. Pieraccini 6, 50139 Florence, Italy,* [3]*Department of Experimental and Clinical Biomedical Sciences, University of Florence, Viale Morgagni 50, 50141 Florence, Italy.*

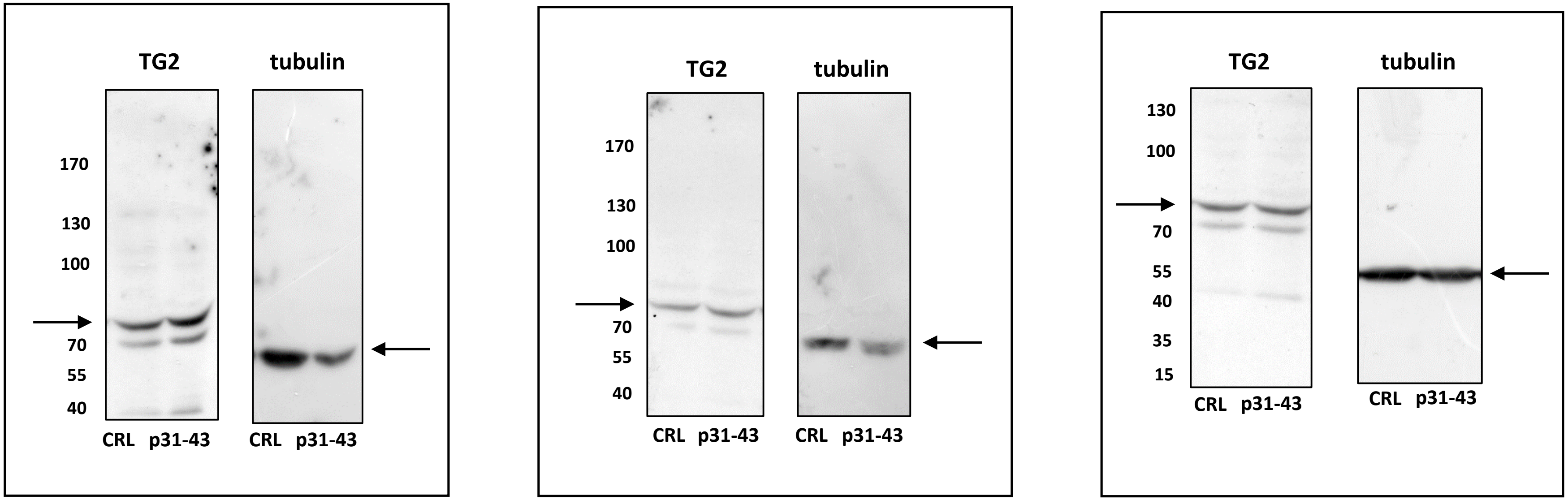

TG2
tubulin
170
130
100
70
55
40
CRL
p31-43
CRL
p31-43
TG2
tubulin
170
130
100
70
55
40
CRL
p31-43
CRL
p31-43
TG2
tubulin
130
100
70
55
40
35
15
CRL
p31-43
CRL
p31-43

Fig. 4a

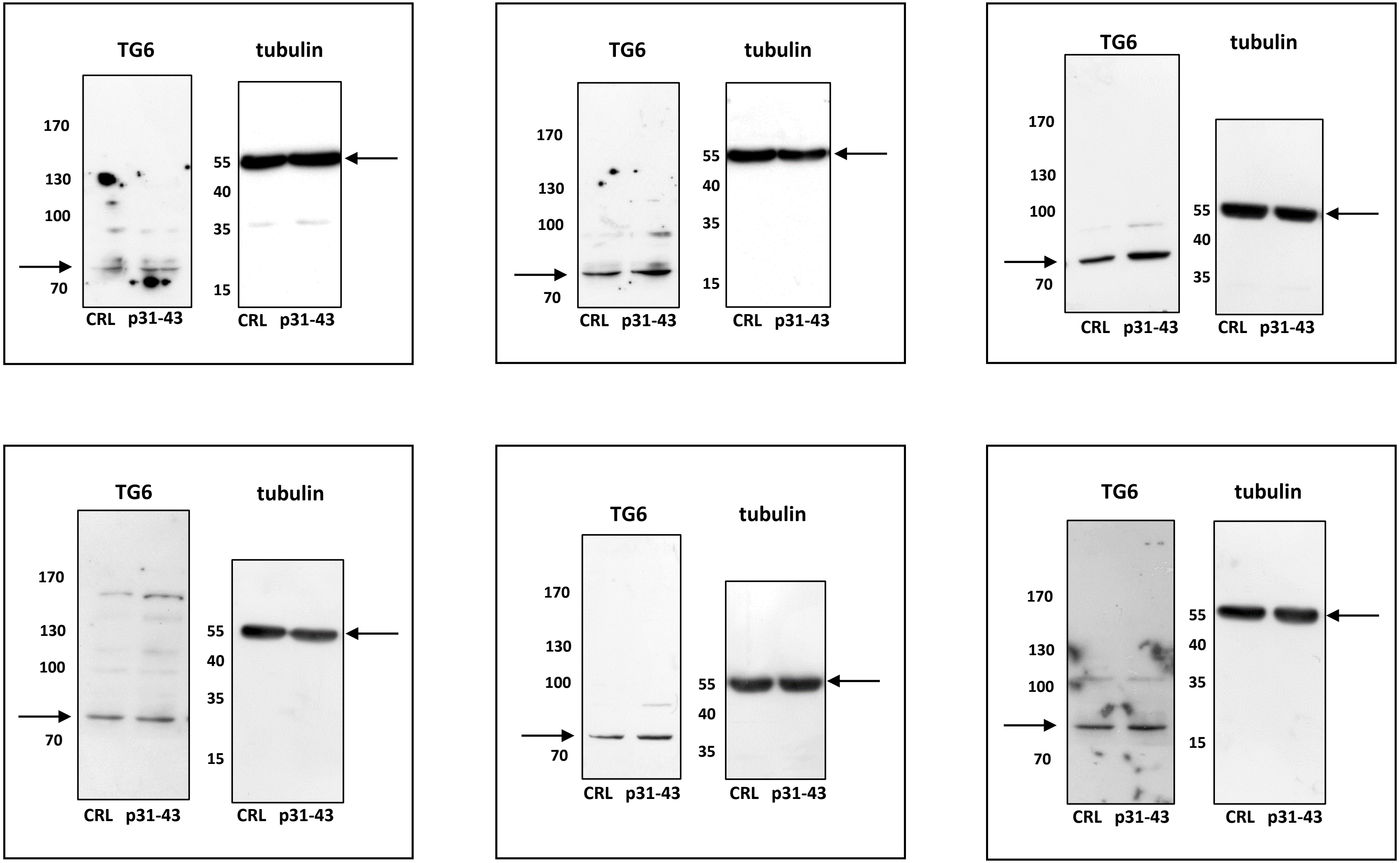
TG6
tubulin
170
130
100
70
55
40
35
15
CRL p31-43
CRL p31-43
TG6
tubulin
170
130
100
70
55
40
35
15
CRL p31-43
CRL p31-43
TG6
tubulin
170
130
100
70
55
40
35
CRL p31-43
CRL p31-43
TG6
tubulin
170
130
100
70
55
40
35
15
CRL p31-43
CRL p31-43
TG6
tubulin
170
130
100
70
55
40
35
CRL p31-43
CRL p31-43
TG6
tubulin
170
130
100
70
55
40
35
15
CRL p31-43
CRL p31-43

Fig. 4b
